# Comparison of machine learning and semi-quantification algorithms for (I123)FP-CIT classification: the beginning of the end for semi-quantification?

**DOI:** 10.1186/s40658-017-0196-1

**Published:** 2017-11-29

**Authors:** Jonathan Christopher Taylor, John Wesley Fenner

**Affiliations:** 1 Nuclear Medicine, Sheffield Teaching Hospitals NHS Foundation Trust, I-floor, Royal Hallamshire Hospital, Glossop road, Sheffield, S10 2JF UK; 2 Insigneo, IICD, University of Sheffield, O-floor, Royal Hallamshire Hospital, Glossop Road, Sheffield, S10 2JF UK

**Keywords:** Parkinson’s disease, 123I-FP, DaTSCAN, Semi-quantification, Machine learning, Support vector machine

## Abstract

**Background:**

Semi-quantification methods are well established in the clinic for assisted reporting of (I123) Ioflupane images. Arguably, these are limited diagnostic tools. Recent research has demonstrated the potential for improved classification performance offered by machine learning algorithms. A direct comparison between methods is required to establish whether a move towards widespread clinical adoption of machine learning algorithms is justified.

This study compared three machine learning algorithms with that of a range of semi-quantification methods, using the Parkinson’s Progression Markers Initiative (PPMI) research database and a locally derived clinical database for validation. Machine learning algorithms were based on support vector machine classifiers with three different sets of features:

- Voxel intensities
- Principal components of image voxel intensities
- Striatal binding radios from the putamen and caudate.

Semi-quantification methods were based on striatal binding ratios (SBRs) from both putamina, with and without consideration of the caudates. Normal limits for the SBRs were defined through four different methods:

- Minimum of age-matched controls
- Mean minus 1/1.5/2 standard deviations from age-matched controls
- Linear regression of normal patient data against age (minus 1/1.5/2 standard errors)
- Selection of the optimum operating point on the receiver operator characteristic curve from normal and abnormal training data

Each machine learning and semi-quantification technique was evaluated with stratified, nested 10-fold cross-validation, repeated 10 times.

**Results:**

The mean accuracy of the semi-quantitative methods for classification of local data into Parkinsonian and non-Parkinsonian groups varied from 0.78 to 0.87, contrasting with 0.89 to 0.95 for classifying PPMI data into healthy controls and Parkinson’s disease groups. The machine learning algorithms gave mean accuracies between 0.88 to 0.92 and 0.95 to 0.97 for local and PPMI data respectively.

**Conclusions:**

Classification performance was lower for the local database than the research database for both semi-quantitative and machine learning algorithms. However, for both databases, the machine learning methods generated equal or higher mean accuracies (with lower variance) than any of the semi-quantification approaches. The gain in performance from using machine learning algorithms as compared to semi-quantification was relatively small and may be insufficient, when considered in isolation, to offer significant advantages in the clinical context.

## Background

(I123) Ioflupane (FP-CIT) or DaTSCAN SPECT imaging is used routinely for evaluation of the function of the striatal dopaminergic pathway. Image interpretation enables differentiation between Parkinsonian and non-Parkinsonian diseases, which may present clinically with similar features. Pooled analysis of phase three and phase four trials showed that (I123)FP-CIT images, when interpreted visually by expert readers, achieved a sensitivity of 88.7% and specificity of 91.2% in the detection of different striatal dopaminergic deficit disorders [1].

In recent years, semi-quantification software, which is intended as an aid to visual reporting, has become commercially available for use in the clinic. In particular, it is recommended by European Association of Nuclear Medicine (EANM) guidelines [2]. Typically, such software provides striatal binding ratios (SBRs) results, which describe the tracer density within small regions of interest as compared to an area of non-specific uptake. These figures give an objective measure of dopaminergic function and give an insight into the likelihood of disease being present. Several studies have suggested that the addition of semi-quantification can improve reporting performance, particularly in terms of reduced equivocal reporting rates and improved inter-observer variability [3–8].

However, semi-quantification is a relatively limited tool for interpreting and classifying (I123)FP-CIT images into different diagnostic groups. Information related to the shape and particular pattern of striatal uptake, which may be important for diagnosis, is not reflected in the SBR results. The figures produced may also be highly dependent on the accuracy of the image registration used, particularly if tight, sub-striatal regions of interest are applied. Semi-quantification software typically produces multiple SBR results from different brain regions, alongside associated normal ranges. The clinician must interpret each SBR result, in light of the normal ranges, to come to an overall decision on image classification.

These shortcomings can potentially be overcome through machine learning algorithms, which can receive multiple input variables describing different features to produce a single metric, such as a probability value, relevant to image classification. Table 8 summarises the available literature on machine learning algorithms for binary (I123)FP-CIT classification (i.e. normal vs abnormal image appearances) since 2010, listed in order of reported maximum accuracy figures, where available. Algorithms using only (I123)FP-CIT SPECT data are considered, multimodal inputs are excluded. The range of approaches adopted by different researchers is wide and varied. However, some general trends can be seen. Support vector machine (SVM) classifiers are the most commonly used algorithms, perhaps because this was considered state-of-the-art until relatively recently (deep learning algorithms now dominate the machine learning literature). The image features tested are relatively simple in the majority of cases. Raw voxel intensities and striatal binding ratios are cited in multiple publications, even in those towards the top of the performance rankings. The Parkinson’s Progression Markers Initiative (PPMI) database of images (www.ppmi-info.org/data) is also frequently cited in validation results, which highlights the fact that many of the reported findings are applicable to research data, not necessarily clinical images.

A range of validation data is used in the publications in Table 8, with different validation methods, some of which are likely to be more biased than others. Results are therefore not directly comparable. However, despite these limitations, the results indicate that machine learning can potentially offer very high levels of performance.

If used as a reporting aid, rather than as a replacement for a radiologist, these tools may offer greater clinical benefits than conventional semi-quantification. However, before moving to clinical trials of machine learning approaches, it would be prudent to compare performance with semi-quantification methods. To the authors’ best knowledge, no direct in-depth comparison has been conducted so far between semi-quantification approaches and machine learning algorithms. Without such evidence, it is difficult to justify investment in clinical translation, and machine learning for (I123)FP-CIT may remain within the province of research, never reaching the clinic.

To aid such justification, this work compares the classification performance of three previously described machine learning approaches with that of a wide range of semi-quantification methods. Classification is considered as a binary task, distinguishing between ‘normal’ and ‘abnormal’ (I123)FP-CIT uptake patterns. Although it is not feasible to test every type of published machine learning algorithm, results presented do provide a baseline comparison to demonstrate whether classical machine learning tools are already sufficiently mature to justify further clinical evaluation.

This study uses two different databases for testing, namely the PPMI database and a local clinical database, from Sheffield Teaching Hospitals NHS Foundation Trust. The PPMI database is relatively large, having the advantage of prospectively recruited healthy and diseased patients, with images acquired on calibrated scanners. It is freely available to researchers and so ensures that results can be directly compared with other algorithms created by different institutions (such as those highlighted in Table 8). Additionally, the use of a local hospital database exercises these methods in an environment that is more relevant to the clinic where diagnostic decisions are made between diseases related to pre-synaptic dopaminergic deficit (PDD) and those unrelated to PDD, rather than between Parkinson’s disease and healthy patients.

## Method

### Data (images and striatal binding ratios)

All screening examinations from the PPMI database were downloaded (209 healthy controls, (HC), 448 with Parkinson’s disease (PD)), including data acquired from multiple different centres, using the same acquisition settings (see Table 1). SBRs were derived from figures supplied by the core lab, whose methods are detailed elsewhere [9]. In short, images were reconstructed in HOSEM software (Hermes Medical, Stockholm, Sweden) using eight iterations and eight subsets OSEM with Chang attenuation correction but without scatter correction or resolution modelling. Images were then passed to PMOD software (PMOD technologies, Zurich, Switzerland) for non-rigid registration to the Montreal Neurological Institute (MNI) template (with manual adjustment), before combining eight axial slices and applying regions of interest in 2D in the putamen, caudate and occipital regions. Images and SBRs from each patient were calibrated using a striatal phantom scanned on the same equipment. Importantly, the diseased group only included patients for which the SPECT images had been visually assessed as having features consistent with PD.

**Table 1:** Summary of patient preparation and image acquisition parameters

Parameter	Local database	PPMI database
Administered activity	167–185 MBq	111–185 MBq
Injection-to-scan delay	3–6 h	3.5–4.5 h
Acquisition time	30 min	30–45 min
Acquisition pixel size	3.68 mm	Variable (scanner dependent)
Number of projections	60 per head (over 180^o^)	120 per head (over 360^o^)
Energy window	159 keV ± 10%	159 keV ± 10% and 122 keV ± 10%
Acquisition matrix size	128 × 128	128 × 128

For the local analysis all (I123)FP-CIT, images were downloaded from the archives at Sheffield Teaching Hospitals and anonymised for inclusion in the study. This included data acquired from four different dual-headed gamma cameras (3 GE Infinia and 1 GE Millenium, GE Healthcare, Chicago, USA), using the same acquisition settings (see Table 1). No camera-specific calibration was performed. However, the similarity in the collimators and detectors between systems should ensure that systematic differences between scanners were small. Details on administered activity and injection-to-scan delay are summarised in Table 1 for both the local database and the PPMI database, alongside image acquisition parameters.

Local images were reconstructed using Xeleris software version 2.1 (GE Healthcare, Chicago, USA), with 2 iterations and 10 subsets, as per the local clinical protocol. Neither attenuation nor scatter correction was performed nor resolution modelling. Each dataset was registered to a template using an affine transformation derived from the Sheffield Image Registration Toolkit (ShIRT; [10]). The registration was performed in stages, transforming the whole brain first and then focusing on individual hemispheres. Registration parameters were set through iterative optimisation, using visual analysis and Dice coefficients to compare results. Regions of interest were derived from those used in DaTSCAN neuro analysis in MIM software v6.7.3 (MIM software Inc., Cleveland, USA), propagating to the template space through non-linear registration. These were applied to image data in 3D to derive SBR values.

Diagnosis was based on the image report, which was produced in a group reporting setup with at least two reporters present in each case. The reporters had full access to previous imaging and other clinical information from the referrer. Cases where significant vascular disease or significant artefacts were identified were excluded. In total, 304 images were retained (113 patients without PDD and 191 with PDD) and 17 excluded. Patients were referred with a range of indications but differential diagnosis of Parkinsonian syndrome vs. essential tremor was the most common. Table 2 provides a summary of the patient population demographics for both the local data and PPMI data.

**Table 2:** Summary of patient demographics

Database	Diagnosis	Sex (total male/total female)	Age (years) Mean (standard deviation)
Local	Non-PDD	61/52	68.7 (12.4)
Local	PDD	132/59	68.7 (13.3)
PPMI	HC	73/136	60.8 (11.3)
PPMI	PD	289/159	61.6 (9.8)

These sets of data present different challenges to semi-quantification and machine learning algorithms. Accuracy is likely to be superior for the PPMI dataset as patient diagnosis is well-established through screening, and diseased patients without obvious dopaminergic deficit are excluded. The local clinical database is more heterogeneous with less certain diagnostic information, deliberately limited exclusion criteria and without quantitative calibration between scanners. This is likely to give rise to a wider array of uptake patterns, with more cross over between normal and abnormal groups, suggesting that accuracy will be lower. However, it is the relative performance of semi-quantification and machine learning that is of most interest, rather than absolute results.

### Semi-quantification methods

There is a range of semi-quantification methods described in the literature and used in commercially available tools. These techniques calculate SBRs from regions of interest applied to the full SPECT volume or selected slices, typically after automated registration to a chosen template. In the clinic, results are usually compared with that of a group of ‘normal’ patients, which may be age-matched, as suggested by EANM guidelines [2]. Normal ranges are often calculated using simple statistical measures (for example, mean SBR ± 2 standard deviations). Usually, the limits of the normal ranges are used as a ‘soft’ cut-off, providing an indication of where the limit of normality lies but open to interpretation by the clinician. Some institutions may define a single cut-off between normal and abnormal groups by considering previously collected data from both healthy and diseased individuals and balancing sensitivity and specificity.

In order to provide objective figures on the accuracy of semi-quantification, hard limits must be defined on SBR figures, with rigid rules on overall classification. In this study, it was assumed that any SBR outside a normal limit cut-off would lead to an overall classification of abnormal. All SBRs must be within normal limits for an overall classification of normal. Although most clinicians would not treat semi-quantification results in this rigid manner, such results provide an indication of the accuracy of the software as an aid to clinical reporting. However, its precise influence is ultimately dependent on the reporting clinician.

In this study, two different approaches to defining SBR cut-offs are investigated: normal limits based on training data from normal subjects only and limits based on data from both diseased and healthy populations. This reflects the different ways in which semi-quantification is used clinically. When using data from normal subjects only, limits are set based on different numbers of standard deviations from the mean or based on a minimum SBR value. Without consideration of SBR figures from diseased patients, this is a naïve approach to classification and is unlikely to achieve the best accuracy. For the second approach, using data from both normal and abnormal patient groups, the best cut-off is defined from the optimal operating point on the receiver operator characteristic (ROC) curve, where the highest classification accuracy is achieved.

Only SBRs from individual putamina (with or without caudate results) are considered. It should be noted, however, that due to limitations in SPECT resolution, it is impractical to isolate uptake in the putamen from that of the adjacent globus pallidus. Thus, all results in this work which refer to the putamen are actually based on uptake in the whole lentiform nucleus. The convention of describing combined putamen and pallidum uptake as that of the putamen alone is maintained to ensure consistent terminology with the literature.

Inclusion of other ratios for performance assessment of semi-quantification (such as right to left ratio and caudate to putamen ratio) is likely to increase the chances of type I error and so are excluded from the analysis. The putamen is the region of the brain that often displays the first signs of dopaminergic degeneration so should be the most sensitive SBR value.

Given the natural decline in SBRs with increasing patient age [11], the semi-quantitative methods investigated account for this confounding variable by either limiting the normal comparison set to an age-matched subset of the training data (test patient age ± 5 years), or they perform a linear regression of SBR against age to derive a mean value from the normal population for the particular test case. The different semi-quantification approaches are summarised in Table 3, grouped according to the method of defining the SBR cut-off. By testing multiple different approaches with different numbers of SBR values and different comparison sets, a comprehensive evaluation of the potential performance of semi-quantitative software can be established.

**Table 3:** Summary of the semi-quantitative methods investigated. Methods are principally grouped according to the particular technique for defining the SBR cut-off

Semi-quantification method	Comparison data	SBRs considered	Cut-offs defined by
SQ 1	Age-matched normals	Left and right putamen	Mean − 2SD
SQ 2	Age-matched normals	Left and right putamen and caudate	Mean − 2SD
SQ 3	Age-matched normals	Left and right putamen only	Mean − 1.5SD
SQ 4	Age-matched normals	Left and right putamen and caudate	Mean − 1.5SD
SQ 5	Age-matched normals	Left and right putamen	Mean − 1SD
SQ 6	Age-matched normals	Left and right putamen and caudate	Mean − 1SD
SQ 7	Age-matched normals	Left and right putamen	Minimum
SQ 8	Age-matched normals	Left and right putamen and caudate	Minimum
SQ 9	All normals	Left and right putamen	Linear regression − 2SE
SQ 10	All normals	Left and right putamen and caudate	Linear regression − 2SE
SQ 11	All normals	Left and right putamen	Linear regression − 1.5SE
SQ 12	All normals	Left and right putamen and caudate	Linear regression − 1.5SE
SQ 13	All normals	Left and right putamen	Linear regression − 1SE
SQ 14	All normals	Left and right putamen and caudate	Linear regression − 1SE
SQ 15	All normals and abnormals	Lowest putamen	Optimal point on ROC curve
SQ 16	All normals and abnormals	Lowest putamen and lowest caudate	Optimal point on ROC curve
SQ 17	Age-matched normals and abnormals	Lowest putamen	Optimal point on ROC curve
SQ 18	Age-matched normals and abnormals	Lowest putamen and lowest caudate	Optimal point on ROC curve

### Machine learning algorithms

**Fig. 1:**
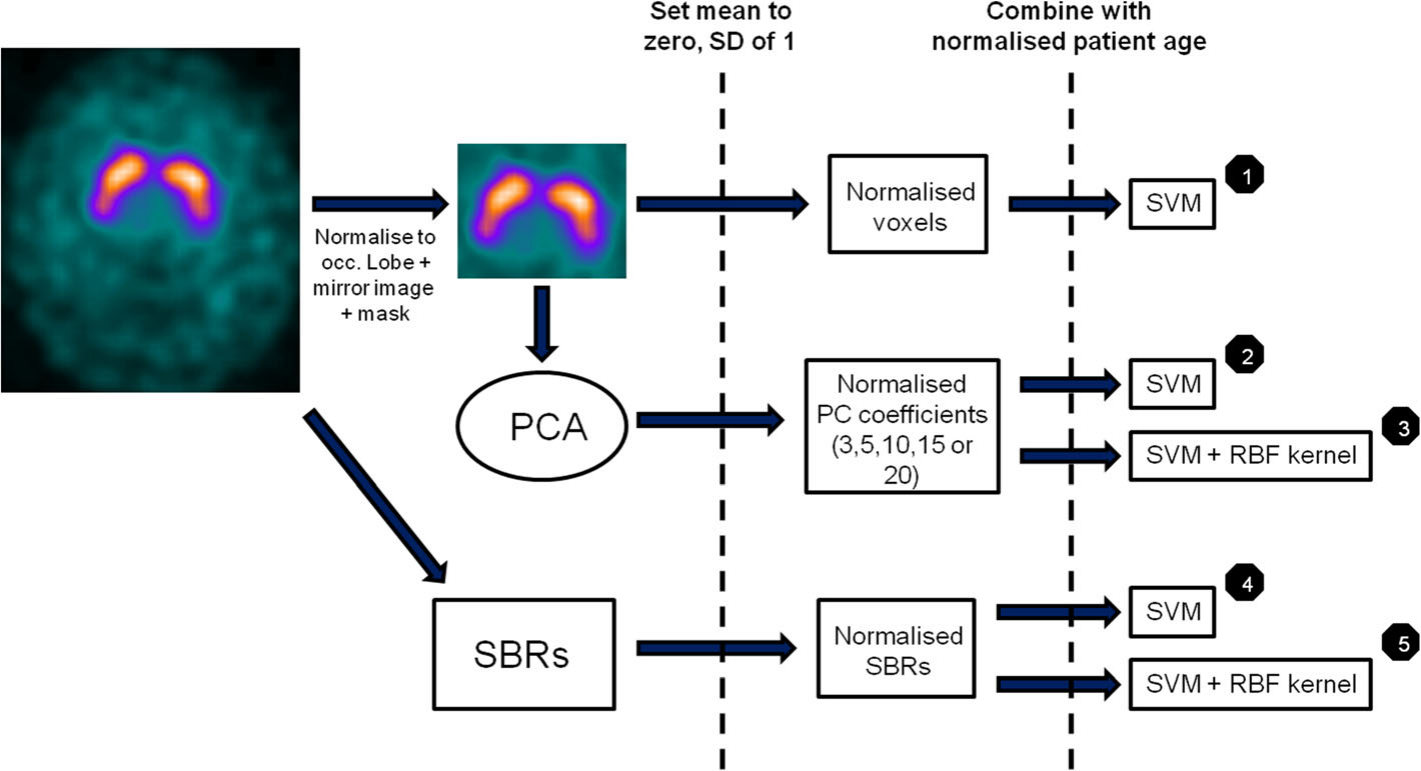
Summary of the machine learning pipelines investigated

In line with general trends seen in Table 8, SVM was used as a classification method, in both conventional linear form and using a radial basis function (RBF) kernel. The simplest image features cited in Table 8 are arguably: image voxel intensities, striatal binding ratios and principal component analysis of image voxels. This study applies these features and classifiers using a pipeline described in Fig. 1. Patient age is used as an added input variable in order to force the classifier to model changes in image appearance with age.

For algorithms taking SBRs as the input, pre-processing involved normalising the binding ratios in each putamen and caudate such that the mean value was zero with a standard deviation of 1. This ensured that each region of uptake was treated with equal importance by the SVM. For the other sets of features, additional pre-processing of the images was first required. Regions of interest were placed over the left and right striata. If necessary, images were flipped about the central axis of the brain to ensure that the most diseased striatum (with the lowest uptake) was always on the left side of the image, as described by Towey et al. [12].

The voxel intensities of each image were scaled to the mean value in the occipital lobe. The central area of the brain, containing the striata was masked with a single, loose region of interest, thus excluding areas that were not considered to be diagnostically important. The remaining normalised voxels or coefficients corresponding to their principal components (either the first 3, 5, 10, 15 or 20 components) were set to a mean value of zero (SD of 1). In the case of features based on voxel values, only a linear SVM was used. Given the very large number of voxel value inputs, the addition of a kernel was unnecessary and could have led to reduced performance due to overfitting. For all other features, both a standard SVM and SVM with RBF kernel were trained and validated.

### Performance comparison

A fair and unbiased comparison between classification techniques is crucial. Classification boundaries should be defined from training data, independent of test data. In this study, each semi-quantitative method and each machine learning algorithm was trained and validated using both sets of clinical databases. A repeated, nested and stratified k-fold cross-validation approach was chosen. This technique splits the available data into different training and test subsections (i.e. different folds) such that classification rules are derived from and applied to different combinations of patient cases. Nesting is used for machine learning algorithms where hyperparameters must be chosen. Here, the training data is further subdivided in order to find the particular combination of hyperparameter values that gives the best accuracy. In this study, a 10-fold cross-validation strategy was chosen. This was repeated 10 times (though not for the inner, nested loops due to limitations in computational resources).

**Fig. 2:**
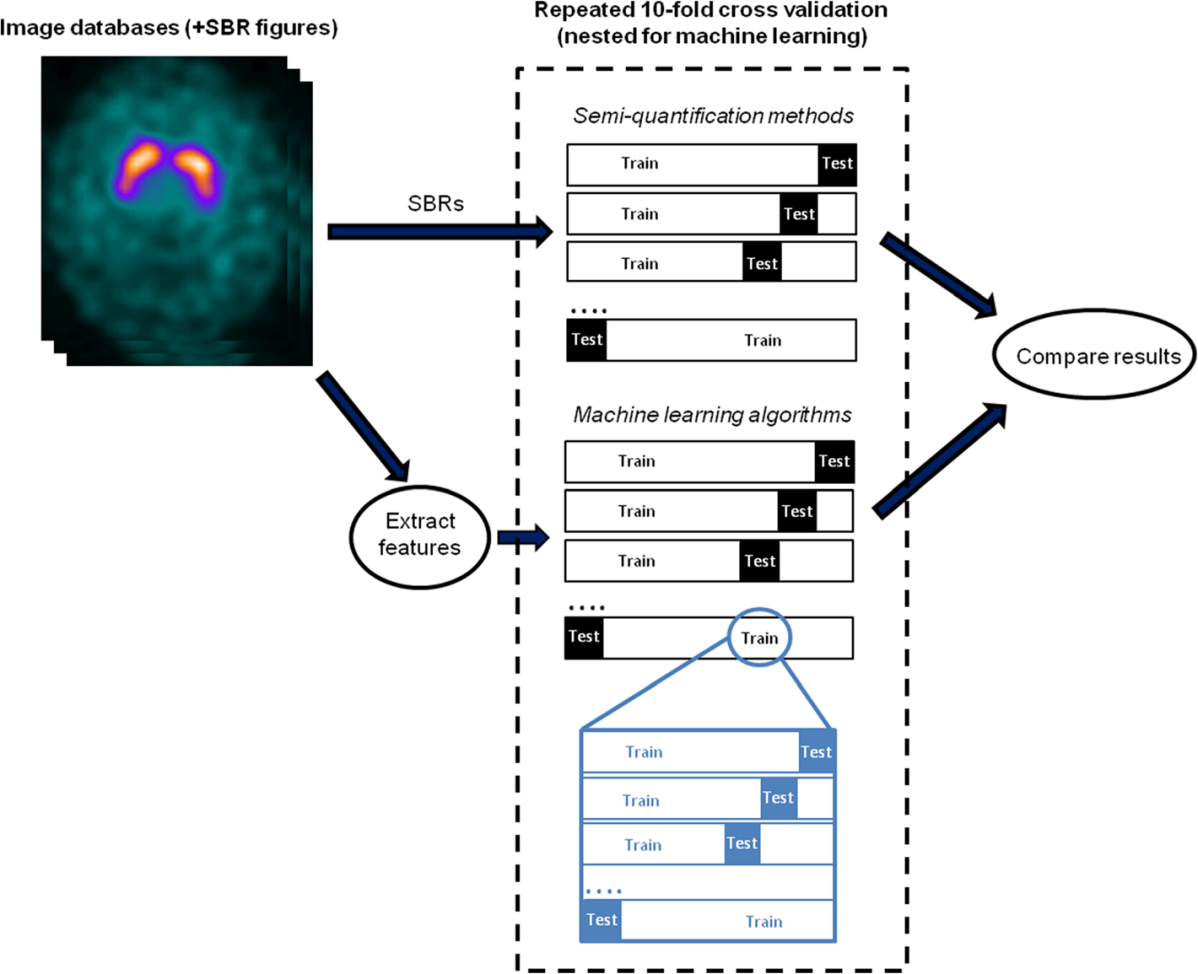
Overview of performance comparison method

All training and testing procedures were carried out with Matlab software (Matlab, Natick, USA), using the libSVM library [13] for defining the SVM classifiers. The hyperparameters of each machine learning algorithm (the ‘C’ regularisation term in the SVM objective function and the gamma term in the RBF kernel) were selected through a coarse grid search in each nested loop. Values between 2^−3^ and 2^8^ were tested for the C parameter and 2^−8^ to 2^3^ for the gamma parameter. The highest mean F-score was used as a metric for selecting the most appropriate values. Figure 2 provides an overview of the testing methodology adopted.

## Results

Tables 4 and 5 show cross-validation results from the semi-quantitative methods, using local and PPMI data respectively. The mean accuracy of the methods for classification of local data varied from 0.78 to 0.87, which as expected was less than that for the PPMI data where mean accuracies varied between 0.89 and 0.95. In general, there appeared to be little influence on performance results when SBR results from the caudate were added to those of the putamen.

**Table 4:** Semi-quantitative results for local clinical data

Method number	Cut-offs defined by	SBRs	Accuracy	SD	Sensitivity	SD	Specificity	SD
SQ 1	Mean − 2SD	L + R putamen	0.79	0.08	0.68	0.12	0.97	0.05
SQ 2	Mean − 2SD	L + R putamen, L + R caudate	0.78	0.08	0.68	0.11	0.96	0.06
SQ 3	Mean − 1.5SD	L + R putamen	0.85	0.06	0.82	0.09	0.90	0.10
SQ 4	Mean − 1.5SD	L + R putamen, L + R caudate	0.85	0.06	0.83	0.08	0.88	0.11
SQ 5	Mean − 1SD	L + R putamen	0.86	0.06	0.91	0.06	0.77	0.12
SQ 6	Mean − 1SD	L + R putamen, L + R caudate	0.86	0.05	0.92	0.06	0.75	0.13
SQ 7	Minimum	L + R putamen	0.83	0.06	0.78	0.08	0.92	0.08
SQ 8	Minimum	L + R putamen, L + R caudate	0.84	0.07	0.81	0.09	0.89	0.10
SQ 9	Regression − 2SE	L + R putamen	0.82	0.07	0.72	0.11	0.99	0.03
SQ 10	Regress − 2SE	L + R putamen, L + R caudate	0.82	0.06	0.72	0.10	0.98	0.04
SQ 11	Regress − 1.5SE	L + R putamen	0.86	0.06	0.82	0.09	0.93	0.09
SQ 12	Regress − 1.5SE	L + R putamen, L + R caudate	0.86	0.06	0.83	0.08	0.91	0.10
SQ 13	Regression − 1SE	L + R putamen	0.87	0.06	0.92	0.06	0.78	0.12
SQ 14	Regress − 1SE	L + R putamen, L + R caudate	0.87	0.06	0.93	0.06	0.77	0.12
SQ 15	ROC age-matched	Lowest putamen	0.87	0.05	0.89	0.06	0.83	0.11
SQ 16	ROC age-matched	Lowest putamen, lowest caudate	0.83	0.07	0.92	0.07	0.67	0.16
SQ 17	ROC	Lowest putamen	0.86	0.06	0.86	0.08	0.86	0.13
SQ 18	ROC	Lowest putamen, lowest caudate	0.84	0.06	0.90	0.07	0.74	0.14

**Table 5:** Semi-quantitative results for PPMI database

Method number	Method	SBRs	Accuracy	SD	Sensitivity	SD	Specificity	SD
SQ 1	Mean − 2SD	L + R putamen	0.93	0.03	0.92	0.04	0.97	0.04
SQ 2	Mean − 2SD	L + R putamen, L + R caudate	0.93	0.03	0.92	0.04	0.96	0.04
SQ 3	Mean − 1.5SD	L + R putamen	0.94	0.03	0.95	0.03	0.92	0.06
SQ 4	Mean − 1.5SD	L + R putamen, L + R caudate	0.94	0.03	0.95	0.03	0.90	0.07
SQ 5	Mean − 1SD	L + R putamen	0.92	0.03	0.98	0.02	0.78	0.09
SQ 6	Mean − 1SD	L + R putamen, L + R caudate	0.89	0.04	0.98	0.02	0.71	0.11
SQ 7	Minimum	L + R putamen	0.90	0.04	0.87	0.05	0.96	0.04
SQ 8	Minimum	L + R putamen, L + R caudate	0.90	0.03	0.88	0.05	0.94	0.05
SQ 9	Regression − 2SE	L + R putamen	0.93	0.03	0.91	0.04	0.97	0.04
SQ 10	Regression − 2SE	L + R putamen, L + R caudate	0.93	0.03	0.91	0.04	0.97	0.04
SQ 11	Regression − 1.5SE	L + R putamen	0.94	0.03	0.95	0.03	0.92	0.05
SQ 12	Regression − 1.5SE	L + R putamen, L + R caudate	0.94	0.03	0.95	0.03	0.90	0.07
SQ 13	Regression − 1SE	L + R putamen	0.92	0.03	0.98	0.02	0.80	0.08
SQ 14	Regression − 1SE	L + R putamen, L + R caudate	0.89	0.04	0.98	0.02	0.71	0.11
SQ 15	ROC age-matched	Lowest putamen	0.94	0.03	0.96	0.03	0.91	0.07
SQ 16	ROC age-matched	Lowest putamen, lowest caudate	0.89	0.03	0.97	0.03	0.73	0.09
SQ 17	ROC	Lowest putamen	0.95	0.03	0.96	0.03	0.92	0.06
SQ 18	ROC	Lowest putamen, lowest caudate	0.89	0.03	0.97	0.03	0.71	0.10

Tables 6 and 7 show cross-validation results from the machine learning methods, using local and PPMI data respectively. Once again, mean accuracies for the local database are lower than that for the PPMI dataset (0.88 to 0.92 and 0.95 to 0.97 respectively). Importantly, every machine learning algorithm exceeded or matched the accuracy results of every semi-quantification method. Standard deviation figures are also smaller than those of the semi-quantification methods in most cases.

**Table 6:** Machine learning results for local clinical data

Method number	Feature	No. PCs	Kernel	Mean accuracy	SD	Sensitivity	SD	Specificity	SD
ML 1	PCs	3	Linear	0.91	0.05	0.93	0.05	0.88	0.10
ML 2	PCs	5	Linear	0.92	0.05	0.94	0.06	0.88	0.10
ML 3	PCs	10	Linear	0.91	0.05	0.93	0.06	0.86	0.10
ML 4	PCs	15	Linear	0.89	0.05	0.92	0.06	0.83	0.11
ML 5	PCs	20	Linear	0.89	0.05	0.92	0.07	0.83	0.12
ML 6	PCs	3	RBF	0.91	0.05	0.91	0.07	0.89	0.09
ML 7	PCs	5	RBF	0.91	0.06	0.92	0.06	0.89	0.10
ML 8	PCs	10	RBF	0.90	0.05	0.91	0.07	0.88	0.09
ML 9	PCs	15	RBF	0.89	0.05	0.91	0.07	0.87	0.10
ML 10	PCs	20	RBF	0.90	0.05	0.90	0.07	0.89	0.10
ML 11	Voxels		Linear	0.88	0.05	0.91	0.06	0.84	0.11
ML 12	SBRs		Linear	0.89	0.05	0.92	0.06	0.82	0.10
ML 13	SBRs		RBF	0.89	0.06	0.91	0.07	0.85	0.10

**Table 7:** Machine learning results for PPMI data

Method number	Feature	No. PCs	Kernel	Mean accuracy	SD	Sensitivity	SD	Specificity	SD
ML 1	PCs	3	Linear	0.97	0.02	0.98	0.02	0.96	0.04
ML 2	PCs	5	Linear	0.97	0.02	0.98	0.02	0.96	0.05
ML 3	PCs	10	Linear	0.97	0.02	0.98	0.02	0.96	0.04
ML 4	PCs	15	Linear	0.97	0.02	0.97	0.02	0.95	0.04
ML 5	PCs	20	Linear	0.97	0.02	0.98	0.02	0.96	0.05
ML 6	PCs	3	RBF	0.97	0.02	0.98	0.02	0.97	0.04
ML 7	PCs	5	RBF	0.97	0.02	0.97	0.02	0.97	0.03
ML 8	PCs	10	RBF	0.97	0.02	0.97	0.02	0.97	0.04
ML 9	PCs	15	RBF	0.97	0.02	0.97	0.02	0.97	0.04
ML 10	PCs	20	RBF	0.97	0.02	0.97	0.02	0.97	0.04
ML 11	Voxels		Linear	0.95	0.02	0.97	0.03	0.92	0.06
ML 12	SBRs		Linear	0.95	0.03	0.97	0.03	0.91	0.06
ML 13	SBRs		RBF	0.95	0.02	0.96	0.03	0.93	0.06

**Fig. 3:**
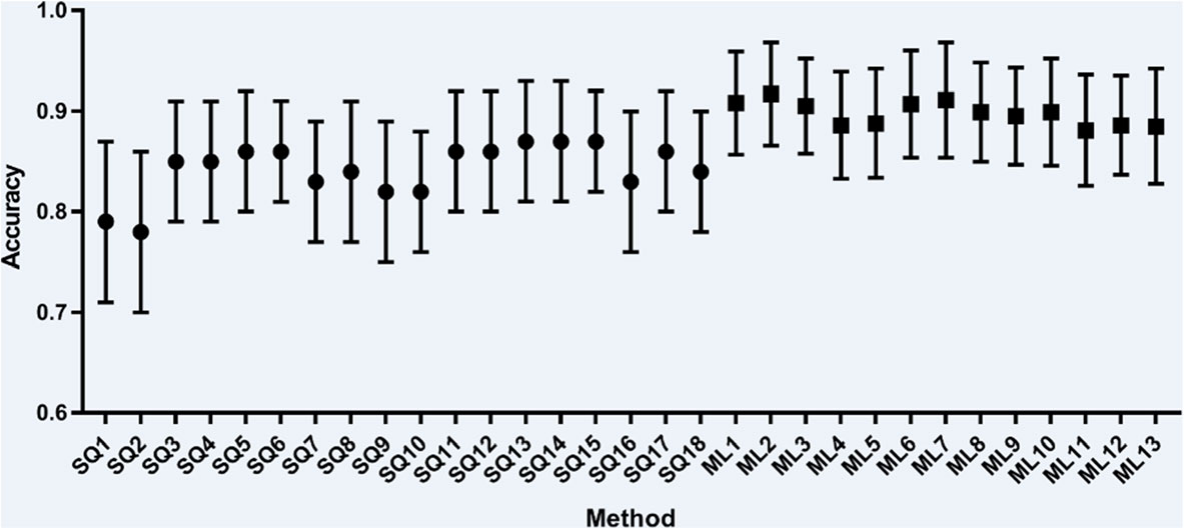
Accuracy results for all semi-quantification and machine learning methods applied to local data. Semi-quantification results are grouped to the left of the graph and machine learning algorithms to the right. Whiskers represent one standard deviation

**Fig. 4:**
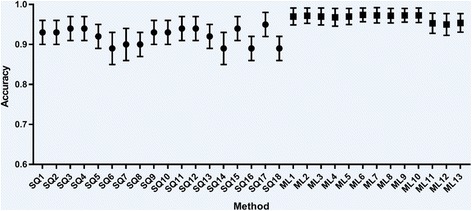
Accuracy results for all semi-quantification and machine learning methods applied to PPMI data. Semi-quantification results are grouped to the left of the graph and machine learning algorithms to the right. Whiskers represent one standard deviation

Figures 3 and 4 summarise the accuracy results of the semi-quantification methods and machine learning algorithms.

## Discussion

This study directly compares the performance of a range of semi-quantification approaches and three machine learning algorithms for classification of (I123)FP-CIT images into normal and abnormal groups. For local data, classification was between patients with pre-synaptic dopaminergic deficit and those without. For the PPMI database, the classification task involved separating patients with Parkinson’s disease from healthy controls. In contrast to much of the literature, the validation method used for comparison was carefully chosen to reduce possible bias. Performing just one iteration of k-fold cross-validation is known to be associated with increased variance [14], and so in this case, the process was repeated 10 times (in the outer validation loops). Stratifying samples in order to maintain similar proportions of normal and abnormal patients in train and test sets has been shown to reduce cross-validation bias [15] and so was also adopted in this study. Nesting the cross-validation, such that any hyperparameter selection was carried out separately in each fold, and with different data to training and testing steps, was also vital for ensuring that bias in performance results was kept to a minimum. This form of validation has been shown to provide an almost unbiased estimate of true classifier error [16].

Clinically, multiple SBRs and other derived ratios may be provided by semi-quantitative software to guide diagnosis. Typically, SBRs from the whole striatum as well as individual caudates and putamina on the left and right side are given. In addition, the caudate to putamen ratio and the right to left ratio may also be displayed. If all these individual SBRs and their associated normal limits are treated as individual tests, the final semi-quantification classification is likely to be overly sensitive (increasing the risk of type I error) and may give a pessimistic view on this form of analysis. Therefore, in this study, only SBRs from individual putamina (with or without caudate results) were considered.

As expected (see Tables 4 and 5), semi-quantification performance was superior for the PPMI dataset as compared to the local clinical database, reaching a maximum accuracy of 0.95 for PPMI and 0.87 for the local data. Variance on performance was also substantially lower for the PPMI data. These differences highlight the substantial difference between performing measurements on well-screened research data acquired according to a rigid protocol with healthy controls and realistic clinical data without an equivalent gold-standard diagnosis and without inter-camera calibration. Results from semi-quantitative evaluation of the local database are similar to those found by other researchers for evaluation of data from a mixed clinical cohort [17], adding confidence to these findings.

Semi-quantitative methods gave a relatively narrow range of accuracy scores across all the methods tested, with a wider range of sensitivities and specificities. Deciding on the ‘best’ performing method depends on the intended application. In clinic for example, a higher specificity than sensitivity may be preferred such that the false positive rate is low. There is no method that stands out in terms of its performance. However, it is interesting to note that two of the methods which treat classification as a two class problem, generating cut-offs from both normal and abnormal putamenal SBRs (i.e. methods SQ 15 and SQ 17), produced some of the highest accuracy figures, with lower variance and well balanced sensitivity and specificity values. This is perhaps unsurprising as all other semi-quantitative methods (which are more reflective of commercially available tools) define cut-offs from the normal population only, with no knowledge of the distribution or likely crossover of abnormal data.

In general, the addition of caudate data to semi-quantitative calculations caused a slight increase in sensitivity and slight reduction in specificity with little effect on accuracy, other than for methods based on ROC curve calculations, which saw a drop in performance. This suggests that the vast majority of diagnostically useful information can be gleaned from consideration of putamen uptake only. Again, this is unsurprising as image appearances often show more marked reduction in the putamen uptake than in the caudate [18].

It is worth noting that the Southampton semi-quantification method [19] was not investigated in this study. Recent research [17] suggests that the sensitivity of this approach is very low when calibration is not performed between different camera systems and is also significantly reduced when correction (including scatter correction) is not performed. Unfortunately, camera-specific calibration data was not available for the local database of images and scatter data were not accessible for the PPMI dataset and so the method was excluded.

The three chosen machine learning approaches are relatively simple and are largely based on previously described algorithms. Undoubtedly, they are not state-of-the-art. In recent years, techniques such as convolutional neural networks have become the dominant technology used by researchers for a range of classification tasks [20]. However, (I123)FP-CIT images have relatively low resolution, with limited variation seen in both normal and abnormal data. Therefore, advanced machine learning techniques may not be necessary to justify consideration for clinical translation. If superior performance can be demonstrated with these classical techniques, then there is a good argument for switching research emphasis from the creation of ever more complex algorithms to clinical evaluation of existing tools.

As shown by Tables 6 and 7 (and Figs. 3 and 4), the machine learning algorithms produced performance metrics that generally exceeded that of the semi-quantitative methods on the same data. All the machine learning algorithms gave accuracies as high as or higher than any of the semi-quantitative methods. Accuracy, sensitivity and specificity were generally high and well balanced for each machine learning tool, with smaller standard deviation values, providing evidence that these approaches are more accurate and less variable than semi-quantification. Machine learning performance metrics for the PPMI data matched the best performing algorithms produced by other authors (see Table 8), with results that are comparable with current state-of-the-art. As with the semi-quantitative results, performance for the PPMI database was substantially higher than for the local data, reinforcing the assertion that classification of the PPMI dataset is a simpler task than that seen in clinical reality.

**Table 8:** Summary of the available literature on machine learning algorithms for binary classification of (I123)FP-CIT images since 2010, ordered according to maximum accuracy (where available)

Authors	Image features	Classifier	Validation data + method	Results
Augimeri et al. 2016 [30]	Mean ellipsoid uptake, dysmorphic index (ellipsoid orientation)	SVM	43 local images (12 normal, 31 Parkinson’s disease (PD)), no cross-validation mentioned	Up to 100% accuracy, specificity and sensitivity
Bhalchandra et al. 2015 [31]	Analysis of 42nd slice only. Striatal binding ratios in both caudates and putamina, radial features and gradient features. Features are tested for statistical significance (wilcoxon rank) before use in the classifier	Linear SVM and SVM with Radial Basis Function (RBF) kernel, Linear Discriminant Analysis (LDA)	350 images from PPMI database (187 healthy controls (HC), 163 PD). 5 fold cross-validation (CV), repeated 100 times	Linear SVM: maximum of accuracy = 99.4% RBF kernel: maximum of accuracy = 99.4% LDA: maximum of accuracy = 99.4%
Oliveira and Castelo-Branco 2015 [32]	Image voxels within striatal region of interest	Linear SVM	654 images from PPMI database (209 HC, 445 PD). Leave-one-out CV	Maximum of accuracy = 97.9% Sensitivity = 97.8% Specificity = 98.1%
Prashanth et al. 2017 [33]	16 shape and 14 surface fitting features of selected slices, following thresholding. Striatal binding ratios of both caudates and putamina and asymmetry indices were also considered. Features are tested for statistical significance (wilcoxon rank) before use in the classifier	SVM with RBF kernel, boosted trees, random forests, naive bayes	715 images from PPMI database (208 HC, 427 PD, 80 scans without evidence of dopaminergic deficit (SWEDD)). 10 fold CV, repeated 100 times. Hyperparameters for SVM chosen through 10 fold CV	SVM: accuracy = 97.3 ± 0.1% Sensitivity = 97.4 ± 0.1% Specificity = 97.2 ± 0.2% Boosted trees: accuracy = 96.8 ± 0.2% Sensitivity = 97.1 ± 0.3% Specificity = 96.3 ± 0.4% Random forests: accuracy = 96.9 ± 0.2% Sensitivity = 97.2 ± 0.2% Specificity = 96.5 ± 0.3% Naive Bayes: accuracy = 96.9 ± 0.1% Sensitivity = 96.4 ± 0.1% Specificity = 96.5 ± 0.2%
Tagare et al. 2017 [34]	Voxel intensities within a region of interest	Logistic lasso	658 images from PPMI database (210 HC, 448 PD). 3 fold CV for performance assessment. Parameters chosen through 10 fold CV (nested within outer 3 fold CV).	Maximum of accuracy = 96.5 ± 1.3%
Palumbo et al. 2014 [35]	Striatal binding ratios for both caudates and putamina (and a subset of these 4 features), patient age	SVM with RBF kernel	90 local images from patients with ‘mild’ symptoms (34 non-PD, 56 PD). Leave-one-out and 5 fold CV	Maximum of accuracy = 96.4%
Prashanth et al. 2014 [36]	Striatal binding ratio for both caudates and putamina	SVM, linear and RBF kernel.	493 images from PPMI database (181 HC, 369 early PD), 10 fold CV, no repeats	RBF kernel: accuracy = 96.1%, sensitivity = 96.6%, specificity = 95.0% Linear SVM: accuracy = 92.3%, sensitivity = 95.3%, specificity = 84.0%
Martinez-Murcia et al. 2013 [37]	12 Haralick texture features within a brain region of interest	Linear SVM	‘Whole’ PPMI database. Leave-one-out CV	Maximum of accuracy = 95.9%, sensitivity = 97.3%, specificity = 94.9%
Zhang and Kagen 2016 [38]	Voxel intensities from a single axial slice, repeated for 3 different slices	Single layer Neural network	1513 images from PPMI database (baseline and follow-up, 1171 PD, 211 HC, 131 SWEDD). 1189 images for training, 108 for validation, 216 for testing. 10 fold CV	Maximum of accuracy = 95.6 ± 1.5%, sensitivity = 97.4 ± 4.3%, specificity = 93.1 ± 3.6%
Rojas et al. 2013 [39]	Voxel intensities, independent component analysis (ICA) & principal component analysis (PCA) decomposition of voxel data (after applying empirical mode decomposition) within regions of interest	Linear SVM	80 local images (39 non-pre-synaptic dopaminergic deficit (non-PDD), 41 PDD). Leave-one-out CV	Raw voxels: accuracy = 87.5%, sensitivity = 90.2%, specificity = 84.6% ICA features: maximum of accuracy = 91.2%, sensitivity = 91.8%, specificity = 92.9% PCA features: maximum of accuracy = 95.0%, sensitivity = 95.1%, specificity = 94.9%
Towey et al. 2011 [12]	PCA decomposition of voxels within striatal region of interest	Naïve-Bayes, Group prototype	116 local images (37 non-PDD, 79 PDD). Leave-one-out CV	Naïve-Bayes: accuracy = 94.8%, sensitivity = 93.7%, specificity = 97.3% Group prototype: accuracy = 94.0%, sensitivity = 93.7%, specificity = 94.6%
Segovia et al. 2012 [40]	Partial least squares decomposition of voxels within striatal regions	SVM applied to hemispheres separately. RBF kernel	189 local images (94 non-PDD, 95 PDD). Leave-one-out CV	Features varied from 1 to 20. Maximum of accuracy = 94.7%, sensitivity = 93.2%, specificity = 93.6%
Martinez-Murcia et al. 2014 [41]	ICA decomposition of selected voxels	SVM, linear and RBF kernel	208 local images (100 non-PDD, 108 PDD), 289 images from PPMI database (114 normal, 175 PD). 30 fold CV	RBF kernel: maximum of accuracy = 94.7%, sensitivity = 98.1%, specificity = 92.0% Linear SVM: maximum of accuracy = 92.8%, sensitivity = 98.2%, specificity = 93.0%
Illan et al. 2012 [42]	Image voxel intensities and image voxels within striatal region of interest	Nearest mean, k-nearest neighbour (k-NN), linear SVM	208 local images (108 non-PDD, 108 PDD). 30 random permutations CV, with 1/3 data held out for testing	SVM: maximum of sensitivity = 89.0%, specificity = 93.2% Nearest mean: maximum of sensitivity = 90.7%, specificity = 84.0% k-NN: maximum of sensitivity = 88.6%, specificity = 86.9%
Palumbo et al. 2010 [43]	Striatal binding ratios for caudate and putamina on 3 slices	Probablistic neural network (PNN), Classification tree (CT)	216 local images (89 non-PDD, 127 PD). Two fold CV, repeated 1000 times	PNN: for patients with essential tremor mean probability of correct classification = 96.6 ± 2.6% CT: for patients with essential tremor mean probability of correct classification = 93.5 ± 3.4%

Algorithms using only (I123)FP-CIT SPECT data are considered, multimodal inputs are excluded. Literature lacking accuracy data are grouped at the bottom of the table

For both databases, algorithms using different numbers of principal components as features gave the highest accuracies (methods ML 1 to ML 10), though the addition of larger numbers of principal components and the use of a non-linear RBF kernel appeared to have little additional impact on results. Although this study considered three principal components as a minimum, preliminary work using just one or two principal components demonstrated relatively high performance figures: mean accuracies (and standard deviations) of 0.87 (0.03), 0.96 (0.02) for linear SVM algorithms trained on PPMI data, using one and two PCs respectively, and mean accuracies of 0.86 (0.06) and 0.89 (0.06), for linear SVM algorithms trained on local data, using one and two PCs respectively. Taken together, these results imply that linear separation between groups can be achieved with very limited numbers of variables.

Features based on raw voxel values and SBRs gave slightly lower performance values in general, more so for the PPMI data. Using voxel intensities as a direct input to a classifier dictates that the problem is ill-posed (due to the very large number of voxel values in comparison to the number of training images). Even with regularisation, performance may be still be affected by over-fitting, which may explain the slightly reduced accuracy. Classifiers based on SBRs are likely to suffer from limitations that are similar to that of semi-quantitative methods, in particular, that information on uptake patterns or striatal shape is lost.

Although machine learning algorithms appeared to perform better than the semi-quantification tools, the clinical context needs to be understood in order to appreciate the significance and value of the results. Firstly, the level of classification performance improvement offered by the machine learning tools is relatively small in this study. It is difficult to determine whether differences were statistically significant due to the re-use of data in each test run. However, examination of the standard deviation on performance results (see Figs. 3 and 4) suggests that there is some crossover in accuracy of the machine learning and semi-quantitative methods. Given that standalone semi-quantification accuracy is approximately 87% for clinical data (and 95% for research data), the margin available for performance gains is real but narrow. Even with the introduction of more advanced tools, there cannot be a substantial gain in accuracy over the algorithms presented here.

Considering that (I123)FP-CIT is a low volume test, used on relatively few clinical patients, the investment required to develop a new clinical reporting tool and pass necessary regulatory hurdles (such as CE marking) may not be commercially justified. In addition, standalone classification performance is a relatively narrow and limited measure of clinical utility. In addition to being untested with radiologists in a realistic reporting scenario, the machine learning classifiers presented here, in common with most of the literature, only provide a decision score as to whether an image is likely to be abnormal or not. Localisation information, providing an indication of the location of any potential abnormalities, is not usually given. This contrasts with semi-quantification approaches which usually provide data on the quadrant(s) of the striata that is (are) affected, which may also be useful for determining the disease subtype. Furthermore, semi-quantification lends itself to use in research as a simple means of grading the severity of disease in response to an intervention. Although machine learning could achieve similar goals (see for example [21]), this aspect of 123I-FP imaging is usually considered as a separate problem.

However, machine learning can offer other benefits. Firstly, these algorithms simplify the information that is shown to the clinician. Rather than having to examine and interpret multiple SBR results and other ratio data, along with their normal ranges, clinicians are presented with a single number representing the overall likelihood of abnormality. Semi-quantification figures are known to be substantially influenced by factors such as the acquisition hardware and reconstruction parameters used [22–27], dictating that normal databases are often acquired separately by individual hospitals. It is possible that machine learning algorithms may be more robust to differences between hospital equipment and protocols, particularly if derived features such as striatal shape are used as input. More work is needed to verify the extent to which such benefits are realisable, which may augment the advantages offered by small increases in classification performance.

In addition, machine learning algorithms can learn disease patterns from multiple heterogeneous inputs. It is possible that by including patient clinical symptoms or results from other tests, diagnostic accuracy and robustness could be further improved. Furthermore, by learning classification models from subtle image features, it may be possible to distinguish between different Parkinsonian syndrome subtypes, such as multiple system atrophy (MSA) and progressive supranuclear palsy (PSP) from (I123)FP-CIT data. Despite the promising research that has been conducted using multimodality inputs [28] and in distinguishing Parkinsonian subtypes [29], rigorous tests on a range of realistic clinical data are lacking.

Although the gain in raw classification performance offered by machine learning may not be sufficient to justify moving away completely from semi-quantification, the results presented here do justify further exploration of machine learning tools. In addition to addressing gaps in our knowledge that have already been mentioned, an interesting avenue of future research would be to combine machine learning and semi-quantification software in such a way as to enhance the information provided to the clinician. In the local context of Sheffield Teaching Hospitals NHS Foundation Trust, the authors will continue to advance machine learning towards the clinic by evaluating the impact of machine learning output on radiologists’ decision-making.

## Conclusions

This study has compared a range of semi-quantification approaches with three selected machine learning methods in order to evidence whether classical machine learning techniques are a superior means of classifying (I123)FP-CIT data into normal and abnormal groups. A research and local clinical database were used for repeated 10-fold cross-validation.

Results showed that classification performance was lower for the local database than the research database for both semi-quantitative and machine learning algorithms. However, for both databases, the majority of the machine learning methods generated higher mean accuracies (with lower variance) than any of the semi-quantification approaches. Mean accuracies for semi-quantification varied from 0.78 to 0.87 for the local database and from 0.89 to 0.95 for the PPMI database. The machine learning algorithms gave mean accuracies between 0.88 to 0.92 and 0.95 to 0.97 for local and PPMI data respectively. In addition, sensitivity and specificity were generally well balanced for the machine learning tools, while they varied more significantly for semi-quantification. This study was performed with machine learning baseline algorithms that can readily be modified for improved performance.

The gain in accuracy from using machine learning algorithms as compared to semi-quantification was relatively small and may not be sufficient to justify a move to exploiting machine learning in the clinical context. A case for clinical translation would have to recognise that machine learning might offer other benefits, such as greater robustness to differences in acquisition conditions.

## Abbreviations

CT: Classification tree
CV: Cross-validation
EANM: European Association of Nuclear Medicine
HC: Healthy control
ICA: Independent component analysis
LDA: Linear discriminant analysis
MNI: Montreal Neurological Institute
PCA: Principal component analysis
PD: Parkinson’s disease
PDD: Pre-synaptic dopaminergic deficit
PNN: Probablistic neural network
PPMI: Parkinson’s Progressive Markers Initiative
RBF: Radial basis function
ROC: Receiver operator characteristic
SBR: Striatal binding ratio
SD: Standard deviation
SE: Standard error
SPECT: Single photon emission computed tomography
SVM: Support vector machine
SWEDD: Scans without evidence of dopaminergic deficit
